# Primary and Secondary Immunodeficiency Diseases in Oncohaematology: Warning Signs, Diagnosis, and Management

**DOI:** 10.3389/fimmu.2019.00586

**Published:** 2019-03-26

**Authors:** Silvia Sánchez-Ramón, Arancha Bermúdez, Luis Ignacio González-Granado, Carlos Rodríguez-Gallego, Ana Sastre, Pere Soler-Palacín, the ID-Signal Onco-Haematology Group, Luis Allende

**Affiliations:** ^1^Hospital Clínico San Carlos, Madrid, Spain; ^2^Hospital U. Marqués de Valdecilla, Santander, Spain; ^3^Hospital U. 12 Octubre, Madrid, Spain; ^4^Hospital Universitario de Gran Canaria Dr. Negrín, Las Palmas de Gran Canaria, Canary Islands, Spain; ^5^Hospital U. La Paz, Madrid, Spain; ^6^Hospital U. Vall d'Hebron, Barcelona, Spain

**Keywords:** immunoglobulins/deficiency, antibodies/deficiency, immunoglobulins/administration and dosage, autoimmunity, hematologic neoplasms, immunologic deficiency syndromes

## Abstract

**Background:** Immunodeficiencies (ID), in particular primary immunodeficiencies (PID), are often associated with haematological manifestations, such as peripheral cytopenias or lymphoproliferative syndromes. Early diagnosis and management have significant prognostic implications. Secondary immunodeficiencies (SID) may also be induced by oncohaematological diseases and their treatments. Haematologists and oncologists must therefore be aware of the association between blood disorders and cancer and ID, and be prepared to offer their patients appropriate treatment without delay. Our aim was to define the warning signs of primary and secondary IDs in paediatric and adult patients with oncohaematological manifestations.

**Methods:** A multidisciplinary group of six experts (2 haematologists, 2 immunologists, and 2 paediatricians specializing in ID) conducted a literature review and prepared a document based on agreements reached an in-person meeting. An external group of 44 IDs specialists from all over Spain assessed the document and were consulted regarding their level of agreement.

**Results:** This document identifies the haematological and extra-haematological diseases that should prompt a suspicion of PIDs in adults and children, in both primary care and haematology and oncology departments. Cytopenia and certain lymphoproliferative disorders are key diagnostic pointers. The diagnosis must be based on a detailed clinical history, physical exploration, complete blood count and standard laboratory tests. The immunological and haematological tests included in the diagnostic process will depend on the care level. Patients who are candidates for immunoglobulin replacement therapy must be carefully selected, and treatment should be offered as soon as possible to avoid the development of complications. Finally, this document recommends procedures for monitoring these patients.

**Conclusions:** This document combines scientific evidence with the opinion of a broad panel of experts, and emphasizes the importance of an early diagnosis and treatment to avoid complications. The resulting document is a useful tool for primary care physicians and specialists who see both adult and paediatric patients with oncohaematological diseases.

## Introduction

Immunodeficiencies (IDs) are a group of diseases caused by quantitative and/or functional changes in the different mechanisms involved in both the innate and the adaptive immune response (1, 2). They are classified as primary immunodeficiency diseases (PIDs) if their origin is genetic, and secondary (SIDs) if their origin is acquired. Both types of IDs are associated with or predispose towards complications, such as infections, autoimmune disorders, immune dysregulation with lymphoproliferation, inflammatory disorders, lymphomas, and other types of cancer, many of which are diagnosed and treated in haematology and oncology departments. PIDs comprise a heterogeneous group of around 400 diseases (3). The Primary Immunodeficiencies Classification Committee of the International Union of Immunology Societies (IUIS) identified 8 large groups of PIDs (9 if phenocopies are included), depending on the underlying immune disorder or the predominant symptom, the most frequent being antibody deficiencies, well-defined syndromes and phagocyte function defects (3). SID, in contrast, is the result of systemic disorders [including haematological disorders, such as chronic lymphocytic leukaemia [CLL]], drugs (e.g., chemotherapeutic agents) and long-term critical or severe diseases (4), that often occur concomitantly in a single patient.

The main haematological manifestations associated with PIDs and SIDs are peripheral cytopenias and immunological dysregulation syndromes (5, 6). These disorders are of particular relevance since they are common in clinical practice, but in many cases they are not listed in the accepted compendia of warning signs for the diagnosis of IDs in children and adults, increasing the risk of failing to diagnose the underlying immunological defect.

Healthcare professionals who see patients with IDs must be aware of these manifestations, so that early diagnosis can be made and treatment can be started promptly. Improving awareness is a crucial step in preventing underdiagnosis and delayed diagnosis, and can help avoid complications (7, 8), improve patient prognosis, lessen the impact on the family, and reduce the social and economic burden of the disease (9). To achieve these aims, specific consensus documents directed at healthcare professionals who see patients with IDs are needed. This document reports the main conclusions of a wide range of specialists on the diagnosis and management of PIDs with haematological manifestations and SIDs associated with oncohaematological disease, and the treatment of these disorders. Our objective was to foster early diagnosis and appropriate treatment of this population. This study is part of the ID-Signal Project, which aims to produce a series of documents describing the clinical manifestations of IDs as they affect the different body systems. The first of this series is a recently published paper on IDs associated with respiratory diseases (10), and other forthcoming documents will focus on rheumatology and neurology.

## Material and Methods

A multidisciplinary group of experts formed of 2 haematologists, 2 immunologists, and 2 paediatricians specializing in IDs identified the issues to be addressed. A review of the literature was then performed, and this review acted as the basis for an in-person discussion of the items to be included in the document. The main conclusions and recommendations were then forwarded to an external panel of experts for their individual evaluation, depending on their speciality. The external panel consisted of 44 experts from all over Spain, with experience in the management of ID, working in Spanish National Health System centres: 14 paediatricians specializing in immunodeficiencies, 17 immunologists who see adult patients and/or perform immunological laboratory assessments of children and adults with suspected ID, 6 paediatricians specializing in haematology and 7 haematologists who see adult patients. This panel indicated their level of agreement on a scale of 1–4, with 1 reflecting “strongly disagree” and 4 “strongly agree.” The results were pooled and the percentages of votes in the categories 1 and 2 (disagreement) and in the categories 3 and 4 (agreement) were calculated. Unanimity was defined as 100% of the experts agreeing with the recommendation/conclusion; consensus, when at least 80% of the experts agreed without unanimity; majority, when >65% and <80% of the experts agreed with the recommendation/conclusion; and disagreement, when the percentage of agreement was 65% or less.

## Results and Discussion

### Haematological Signs That Should Prompt a Suspicion of PID

To date, the causative genetic defect has been identified in 344 of the different PIDs (3). The IUIS classification divides these PIDs into 9 categories (3). At present, autoimmunity and dysregulation of the immune system are thought to be the underlying causes of a growing number of PIDs that typically manifest initially as cytopenias. In PIDs specifically, cytopenia may be caused by cellular or humoral autoimmunity, immune dysfunction in the form of hemophagocytosis or lymphoproliferation with or without splenic sequestration, bone marrow failure with myelodysplasia or secondary myelosuppression (11). Table 1 shows the haematological signs or manifestations that, according to the panel of experts, should prompt suspicion of PIDs in primary care and haematology consultations, differentiating between paediatric and adult patients. The panel also agreed on the common haematological manifestations that might appear in each of the PIDs categories (Table 2). Some extra-haematological diseases, such as skin manifestations, gastrointestinal disorders, recurrent pneumonia, or growth retardation, among others, should also be taken into consideration.

**Table 1:** Haematological manifestations that should prompt a suspicion of PID in primary care and haematology consultations.

	***N* (Composition of panel)**	**Votes in agreement (%)**	**Degree of agreement**
**PRIMARY CARE**			
**Adult patients**			
Recurrent neutropenia	24 (AI, AH)	95.8	**Consensus**
Persistent thrombocytopenia	24 (AI, AH)	75.0	Majority
Persistent lymphocytopenia	24 (AI, AH)	100.0	**Unanimity**
Lymphoproliferation: lymphadenopathies and hepatosplenomegaly	24 (AI, AH)	95.8	**Consensus**
**Paediatric patients**			
Recurrent neutropenia	20 (PI, PH)	100.0	**Unanimity**
Persistent thrombocytopenia	20 (PI, PH)	95.0	**Consensus**
Persistent lymphocytopenia	19^*^ (PI, PH)	100.0	**Unanimity**
Lymphoproliferation: lymphadenopathies and hepatosplenomegaly	19^*^ (PI, PH)	100.0	**Unanimity**
Lymphocytopenia in infancy	19^*^ (PI, PH)	100.0	**Unanimity**
Persistent neutropenia	19^*^ (PI, PH)	100.0	**Unanimity**
**HAEMATOLOGY CLINICS (ADDITIONAL HAEMATOLOGICAL MANIFESTATIONS)**			
**Adult and paediatric patients**			
Bone marrow failure: aplasia, myelodysplasia and myelokathexis	44 (AI, PI, AH, PH)	86.4	**Consensus**
Persistent leukocytosis without malignancy or infection: neutrophilia, eosinophilia, lymphocytosis	44 (AI, PI, AH, PH)	79.5	Majority
**Paediatric patients**			
Same as for adults	18^*^ (PI, PH)	100.0	**Unanimity**
Lymphocytopenia in infancy	19^*^ (PI, PH)	100.0	**Unanimity**

*AI, adult immunologists; AH, adult haematologists; PC, primary care; PH, paediatric haematologists; PI, paediatric immunologists; PID, primary immunodeficiency disease*.

* *Some missing values. “Persistent” is defined as duration of more than 1 month with no obvious cause. Unanimity: 100% of the experts agreeing with the recommendation/conclusion. Consensus: at least 80% of the experts agreed without unanimity. Majority: when more than 65% and less than 80% of the experts agreed with the recommendation/conclusion. Disagreement: percentage of agreement was 65% or less*.

**Table 2:** Common haematological manifestations by PID type.

**COMMON HAEMATOLOGICAL MANIFESTATIONS BY PID TYPE**	**Votes in agreement (%)**	**Degree of agreement**
**COMBINED ID**		
Immune cytopenias	88.6	**Consensus**
Non-immune cytopenia: lymphopenia	93.2	**Consensus**
Lymphoproliferative disorders associated with viral infections	95.5	**Consensus**
Aplasia/myelodysplasia/myelokathexis	90.9	**Consensus**
Eosinophilia	81.8	**Consensus**
Lymphocytosis	72.7	Majority
**ANTIBODY DEFICIENCIES**		
Immune cytopenias	97.7	**Consensus**
Non-immune cytopenia: thrombocytopenia	77.3	Majority
Lymphoproliferative disorders associated with viral infections	79.5	Majority
**IMMUNE DYSREGULATION**		
Immune cytopenias	100.0	**Unanimity**
Lymphoproliferative disorders associated with viral infections	95.5	**Consensus**
Eosinophilia	81.8	**Consensus**
Lymphocytosis	79.5	Majority
**PHAGOCYTE DISORDERS**		
Non-immune cytopenia: neutropenia	97.7	**Consensus**
Non-immune cytopenia: monocytopenia	90.9	**Consensus**
Aplasia/myelodysplasia/myelokathexis	79.5	Majority
Neutrophilia	81.8	**Consensus**
**INNATE IMMUNITY DISORDER**		
Non-immune cytopenia: neutropenia	93.2	**Consensus**
**AUTOINFLAMMATORY DISEASE**		
Non-immune cytopenia: neutropenia	72.7	Majority
Neutrophilia	88.6	**Consensus**

*AI, adult immunologists; AH, adult haematologists; ID, immunodeficiency; PH, paediatric haematologists; PI, paediatric immunologists; PID, primary immunodeficiency disease*.

*N (Composition of panel), 44 (AI, PI, AH, PH) for all manifestations. Unanimity: 100% of the experts agreeing with the recommendation/conclusion. Consensus: at least 80% of the experts agreed without unanimity. Majority: when more than 65% and less than 80% of the experts agreed with the recommendation/conclusion. Disagreement: percentage of agreement was 65% or less*.

### Diagnostic Approach When PID Is Suspected

Most PIDs are diagnosed with a complete, targeted clinical history, detailed physical examination, complete blood count, and standard laboratory tests, including the determination of serum immunoglobulin (Ig) levels. The clinical history should include family history of PID, consanguinity or family history of sudden death at an early age. Physical examination should include an assessment of nutritional status, sequelae from previous infections, lymph nodes, tonsils, hepatosplenomegaly, etc. A complete blood count and blood smear will rule out cytopenia or cell abnormalities (12). These basic laboratory tests should also include liver and kidney function, total protein and albumin. Determination of serum Ig (IgG, IgM, IgA, and IgE) is the first step in the evaluation of humoral immunity, and will help diagnose quantitative Ig deficiencies, such as congenital agammaglobulinemia, common variable ID, or IgA deficiency, and other antibody abnormalities associated with defects such as hyper-IgE or hyper-IgM syndrome (12).

It is important to assess the results according to the reference values for each age, since there are significant differences which, if not taken into account, can lead to the PID being overlooked or also giving a wrong diagnosis of PID (13, 14). When diagnosis remains uncertain after all these tests and suspicion is high, additional tests, such as functional or molecular studies, must be performed in reference centres. Table 3 specifies the additional immunological and haematological tests, according to care level, and in the case of immunological tests, according to the type of PID suspected. Although all the immunological tests reached consensus (Table 3), some important proposals were made that the expert panel agreed to include separately from the table. In terms of haematological assessment, cytological studies of bone marrow aspirate and biopsy specimens are strongly recommended (11). Bone marrow and/or tissue immunophenotyping is recommended, depending on the disease under consideration, for example, in the case of lymphoproliferative disease. Finally, cytogenetic studies are recommended in patients with bone marrow failure.

**Table 3:** Additional to baseline immunological and haematological tests in patients with suspected PID.

**Baseline level**	**First level^**a**^**	**Second level^**b**^**	**Third level^**b**^**	Votes in agreement (%)^**^
**ADDITIONAL TO BASELINE IMMUNOLOGICAL TESTS**				
Combined^*^				
Complete blood count IgA, IgG, IgM, and IgE levels Biochemical analysis	Lymphocyte populations	Extended phenotype, lymphocyte function	Protein expression Functional and genetic studies	86.7
PID-associated syndromes^*^				
Complete blood count IgA, IgG, IgM, and IgE levels Biochemical analysis	Karyotype ± CGH Array	Study according to specific suspicion	Protein expression Functional and genetic studies	86.7
**Antibody production deficiency**				
Complete blood count IgA, IgG, IgM, and IgE levels Biochemical analysis	Basic antibody production study (ASLO, hemaglutinins and tetanus)^***^	IgG subclasses Response to pneumococcus or *Salmonella typhi*. Response to tetanus toxoid and *H. influenzae* (optional) Lymphocyte populations with extended phenotype B	Protein expression Functional and genetic studies	90.3
Immune dysregulation^*^				
Complete blood count IgA, IgG, IgM, and IgE levels Biochemical analysis	Autoantibody panel (ANA and NOSAB, anti-neutrophils) Targeted hormone study Coombs test Vitamin B12 with DNT Soluble FAS ligand Ferritin Triglycerides Fibrinogen Calcium and phosphorus	Treg Soluble CD25 FoxP3	Protein expression Functional and genetic studies	96.7
**Phagocyte disorders**				
Complete blood count IgA, IgG, IgM, and IgE levels Biochemical analysis	Oxidation test (DHR) Basic lymphocyte subpopulations	CD18 and CD11b	-	90.3
**Innate immunity disorder**				
Complete blood count IgA, IgG, IgM, and IgE levels Biochemical analysis	Studies according to clinical suspicion	-	Protein expression Functional and genetic studies	96.8
**Complement deficiency**				
Complete blood count IgA, IgG, IgM, and IgE levels Biochemical analysis	CH50, C3, C4, autoimmunity studies	AP50 Individual factor quantification	Protein expression Functional and genetic studies	93.5
**Autoinflammatory diseases**				
Complete blood count IgA, IgG, IgM, and IgE levels Biochemical analysis	Inflammatory markers CRP ESR	SAA	Genetic studies	93.5
		**Second and third level**^**b**^	**Fourth level**^**c**^	Votes in agreement (%)^**^
**HAEMATOLOGICAL TESTS**				
		Peripheral blood smear Bone marrow aspirate/biopsy	Telomere length study Flow cytometry analysis of DNA repair TCR and IgH rearrangements	85.7

*ANA, Antinuclear antibodies; NOSAB, non-organ specific autoantibodies; ASLO, Antistreptolysin O; COOMBS, antiglobulin test; CRP, C-reactive protein; DHR, dihydrorhodamine 123; DNT, double negative alpha/beta T cells; ESR, erythrocyte sedimentation rate; SAA, Serum amyloid A protein; Treg, regulatory T cells*.

a *First-level tests will be carried out in primary care or hospital centres which have laboratories with the necessary resources*.

b *Second and third-level tests will be carried out in reference centres (in haematology unit for haematological tests)*.

c *Fourth-level tests will be carried out by immunodeficiency specialists or in reference centres*.

N and Composition of panel: 31 (30^*^) adult haematologist and paediatric immunologists for additional to baseline immunological tests and 7 adult haematologist for haematological tests.

* *Some missing values*.

** *Degree of agreement in all cases: Consensus*.

*** *If available*.

In summary, the entire diagnostic process must combine immunological and haematological approaches, especially when cytopenia is the initial manifestation of PID, in order to guide and accelerate the differential diagnosis of a significant number of autoimmune diseases, lymphoproliferative disorders and others (11). This approach will help minimize the damage that might occur if the PID diagnosis is delayed (15).

### Secondary Immunodeficiencies in Oncohaematology

SID is much more common than PID in oncohaematology and in general. The causes of SID in oncohaematology are summarized as follows: i) Systemic disorders: aplastic anaemia, haematological malignancies, such as CLL, multiple myeloma (MM), Hodgkin's disease, non-Hodgkin lymphoma (NHL), graft vs. host disease, and sickle cell disease; ii) Iatrogenic disorders caused by certain drugs (chemotherapy, immunosuppressants, corticosteroids, monoclonal antibodies, such as anti-CD20 agents, and B cell differentiation and maturation inhibitors), radiation therapy, splenectomy and bone marrow ablation before transplantation; iii) Prolonged severe disease, particularly in critically ill, elderly, and hospitalized patients.

These IDs appear clinically as more frequent infections or unusual infective complications and, occasionally, as opportunistic infections (4). Immune system abnormalities induced by SIDs affect both innate and adaptive immunity; they can be subtle and clinical manifestations are often heterogeneous.

In the case of haematological malignancies, such as MM, CLL, and lymphomas, both the underlying disease and the immunosuppressive treatment can contribute in different ways to the emergence of SID.

The scientific consensus committee agreed that the more common haematological conditions and situations that may predispose to SIDs are the following:

- Post-transplantation with partial immune recovery
- CLL
- MM
- NHL
- Treatment with rituximab and new generations of anti-CD20 monoclonal antibodies, alemtuzumab, or combination protocols that include the administration of fludarabine.
- New therapeutic strategies targeting the B cell receptor signalling pathway (e.g., Syk and PI3K enzymes or mTOR transcription factor), the recruitment of cytotoxic B cells [e.g., T cell therapy with chimeric antigen receptor [CAR]], and B cell apoptosis inducers (e.g., navitoclax).

When SID is suspected, basic screening procedures, similar to those performed to rule out PID, should be conducted. This involves a complete blood count, quantification of serum immunoglobulins, and biochemistry panel (basic liver and kidney function, total protein and albumin). Antibody production assessment is also recommended when possible. Additional haematological tests recommended by the panel of experts are listed in Table 4.

**Table 4:** Haematological tests to be performed in patients with suspected SID.

**Baseline level**	**First level^**a**^**	**Second level^**b**^**	**Third level^**b**^**	Votes in agreement (%)^*^
**SID**				
Complete blood count IgA, IgG, IgM, and IgE levels Biochemical analysis	Basic antibody production study (ASLO, hemaglutinins and tetanus)^**^	IgG subclasses Vaccine response to pneumococcus or *Salmonella typhi*. Response to tetanus toxoid and *H. influenzae* (optional) Lymphocyte subpopulations with expanded phenotype B.	-	93.5

*Antistreptolysin O*.

a *First-level tests will be carried out in primary care or hospital centres which have laboratories with the necessary resources*.

b *Second and third-level tests will be carried out in reference centres*.

* *Degree of agreement: Consensus*.

** *If available*.

*N and Composition of panel: 31 both adult and paediatric immunologists*.

### Intravenous and Subcutaneous Immunoglobulin Replacement Therapy for PID and SID With Haematological Manifestations

Immunoglobulin replacement therapy (IGRT) is essential in patients with PIDs and SIDs that directly affect B cell function and antibody production and, in the case of SID, that present with severe or recurrent infections (16). The indications for IGRT continue to expand, as the characteristics, effects, and clinical presentations of IDs become better known.

#### Treatment of Primary Immunodeficiencies

In currently characterized PIDs phenotypes, intravenous, or subcutaneous IGRT is indicated in agammaglobulinemia due to the absence of B cells, and in hypogammaglobulinemia with deficient antibody production. However, the use of IGRT must be individually evaluated in patients with normal IgG and defective antibody function, hypogammaglobulinemia, and normal antibody function, isolated deficiency of IgG subclasses with recurrent infections, and recurrent infections due to complex, uncharacterized immunological mechanisms (17, 18).

#### Treatment of Secondary Immunodeficiencies

The use of subcutaneous or intravenous IGRT for SIDs is less well defined than for PIDs and is mainly based on experience in the treatment of the latter (18, 19). IGRT may be considered in highly selected patients with CLL who also have hypogammaglobulinemia with recurrent bacterial infections and deficient production of specific antibodies, since infections are currently the main cause of morbidity and mortality in these patients. Specific antibodies tests have been assigned to reference centres (second level) because with the exception of anti-tetanus IgG and Antistreptolysin O, other specific antibodies as pneumococcus or *Salmonella typhi*, are usually not available in primary care and even some regional hospitals. Moreover, it seems reasonable, at least in those cases without a clear evidence of primary antibody deficiencies to initially test for IgG levels and measure specific antibodies in a second level. IGRT is also an option in patients with MM and recurrent serious bacterial infections, in patients who have received haematopoietic stem cell transplantation, and in patients with many other diseases (cancer and inflammatory or autoimmune diseases) who have received treatments that deplete B cell levels (16, 20–22). The immunological impact on SIDs of new therapies that target key molecules in the differentiation, maturation or apoptosis of B cells has not yet been explored (23). Experience acquired with the first generation of anti-CD20 monoclonal antibodies has shown that low baseline levels of IgM and IgG in serum are associated with an increased risk of infection and a cumulative effect after repeated cycles has been observed (24). Secondary antibody responses do not seem to be affected following therapy with anti-CD20 monoclonal antibodies, but several immunization studies have shown that primary responses are markedly impaired (25). Recent data suggest that CD19-targeted CAR therapy might preserve patients' humoral immunity (26).

With regard to the choice between subcutaneous or intravenous administration, both routes have shown equivalence in terms of efficacy and safety (17, 27, 28), although systemic adverse effects are more common with the intravenous route, and milder local effects are observed with the subcutaneous route (28–30). Despite the need to train the patient and the requirement for simple devices for proper administration, the subcutaneous route has a series of significant advantages: it is associated with improved quality of life for both patients and their caregivers (28, 31–33); it has been shown to be more cost-effective, mainly due to fewer days missed from work and school (28); and it is a better solution for patients with problems for venous access and mobility issues. Finally, patient preferences are an essential part of the decision-making process (34).

### Monitoring IDs Patients With Haematological Involvement

The number of infections and subsequent hospitalizations has decreased in patients with certain IDs in recent years, thanks to improvements in diagnosis and treatment. However, these patients still require regular clinical monitoring (12). Personalized decisions on the periodicity of monitoring can be made on the basis of the patients' age, clinical status and type of ID. Patients should be assessed by an immunologist at least once every 6–12 months, and a full clinical history, physical examination, complete blood count and biochemistry with LDH and immunoglobulins must be obtained (12). During dose adjustment, IgG values should be determined more frequently. Table 5 summarizes the agreements reached by the expert group.

**Table 5:** Minimum tests to be performed during monitoring of the patient with ID and haematological symptoms.

**TESTS**	***N*(Composition of panel)**	**Votes in agreement (%)**	**Degree of agreement**
Patient monitoring every 6–12 months or in case of evidence of clinical infection	31 (AI, PI)	90.3	**Consensus**
Immunological studies (complete blood count, biochemistry with LDH and Igs) every 6–12 months or in case of infection	31 (AI, PI)	87.1	**Consensus**
In the event of replacement therapy with gammaglobulins, IgG values must be measured more frequently, at least during dose adjustment.	31 (AI, PI)	100.0	**Unanimity**
In case of complete remission of the haematological disease, patient monitoring every 3 months is recommended. Otherwise, the patient should be seen every month, depending on the underlying disease.	30^*^ (AI, PI)	93.3	**Consensus**

*AI, adult immunologists; ID, immunodeficiency; LDH, lactate dehydrogenase; PI, paediatric immunologists*.

* *Some missing values. Unanimity: 100% of the experts agreeing with the recommendation/conclusion. Consensus: at least 80% of the experts agreed without unanimity. Majority: when more than 65% and less than 80% of the experts agreed with the recommendation/conclusion. Disagreement: percentage of agreement was 65% or less*.

## Conclusions

Peripheral cytopenias and several lymphoproliferative disorders should prompt a suspicion of PID. The diagnosis must be based primarily on the clinical history, a detailed physical exploration, a complete blood count and the determination of serum Ig levels. Further immunological and haematological tests included in the diagnostic process will depend on the patient's clinical phenotype and health care level at which they are seen. Immunoglobulin replacement therapy, when indicated, should be offered as soon as possible to avoid complicated infections and organ damage.

This study is the first to focus on both primary and secondary IDs from an oncohaematological perspective. It addresses the diagnosis, treatment and follow-up of both adult and paediatric patients, and reflects the opinion and clinical experience of more than 40 interdisciplinary experts from different fields and specialities who regularly treat these patients. This document is intended to be a useful tool for doctors working in any field who might see patients with ID, to help them to identify the warning signs of an ID associated with haematology. Other approaches to promote the early identification of PIDs and SIDs should include the training of professionals.

## Author Contributions

SS-R and PS-P made substantial contributions to the conception and design of the document, to the first draft of the document (before the evaluation of the external panel of experts), and also to the analysis and interpretation of data from the external panel of experts; they have also been involved in drafting and revising the manuscript critically for important intellectual content and have given final approval of the version to be published. AB, LG-G, CR-G, and AS made contributions to the first draft of the document (before the evaluation of the external panel of experts) and to the interpretation of data from the external panel of experts; they also have been involved in revising the manuscript and have given final approval of the version to be published. ID-Signal Onco-Haematology Group evaluated the conclusions and recommendations individually.

### Conflict of Interest Statement

SS-R has served as speaker, consultant, and advisory member for or has received research funding from CSL Behring, Grifols, Shire, and Octapharma. AB reports personal fees from CSL Behring, outside the submitted work. LG-G reports grants from Fondo De Investigación Sanitaria Carlos III, from Shire, grants and personal fees from CSL Behring, during the conduct of the study. CR-G reports personal fees from CSL Behring, during the conduct of the study, and personal fees from Shire SL outside the submitted work. AS reports personal fees from CSL, during the conduct of the study. PS-P reports personal fees from CSL Behring, grants from Jeffrey Modell Foundation, during the conduct of the study; personal fees from Shire SL, personal fees from Grifols, outside the submitted work.
